# Model-Based Hypothesis Testing of Key Mechanisms in Initial Phase of Insulin Signaling

**DOI:** 10.1371/journal.pcbi.1000096

**Published:** 2008-06-20

**Authors:** Gunnar Cedersund, Jacob Roll, Erik Ulfhielm, Anna Danielsson, Henrik Tidefelt, Peter Strålfors

**Affiliations:** 1 Department of Clinical and Experimental Medicine, Linköping University, Linköping, Sweden; 2 Department of Electrical Engineering, Linköping University, Linköping, Sweden; University of Auckland, New Zealand

## Abstract

Type 2 diabetes is characterized by insulin resistance of target organs, which is due to impaired insulin signal transduction. The skeleton of signaling mediators that provide for normal insulin action has been established. However, the detailed kinetics, and their mechanistic generation, remain incompletely understood. We measured time-courses in primary human adipocytes for the short-term phosphorylation dynamics of the insulin receptor (IR) and the IR substrate-1 in response to a step increase in insulin concentration. Both proteins exhibited a rapid transient overshoot in tyrosine phosphorylation, reaching maximum within 1 min, followed by an intermediate steady-state level after approximately 10 min. We used model-based hypothesis testing to evaluate three mechanistic explanations for this behavior: (A) phosphorylation and dephosphorylation of IR at the plasma membrane only; (B) the additional possibility for IR endocytosis; (C) the alternative additional possibility of feedback signals to IR from downstream intermediates. We concluded that (A) is not a satisfactory explanation; that (B) may serve as an explanation only if both internalization, dephosphorylation, and subsequent recycling are permitted; and that (C) is acceptable. These mechanistic insights cannot be obtained by mere inspection of the datasets, and they are rejections and thus stronger and more final conclusions than ordinary model predictions.

## Author Summary

Insulin is a central player in maintaining energy balance in our bodies and in type 2 diabetes, where the effect of insulin on its target tissues is diminished. Insulin acts on cells by binding to specific insulin receptors (IRs) at the cell surface. This triggers a series of events, including attachment of phosphate to IR, activation of downstream proteins that eventually mediate the signal to specific targets in the cell, and internalization of IR to the inner cytosolic part of the cell. The importance, time relations, and interactions between these events are not fully understood. We have collected experimental time-series and developed a novel analysis method based on mathematical modeling to gain insights into these initial aspects of how insulin controls cells. The main conclusion is that either IR internalization and the subsequent recycling back to the cell surface or feedbacks from downstream proteins (or both) must be significantly active during the first few minutes of insulin action. These conclusions could not have been reached from the experimental data through conventional biological reasoning, and this work thus illustrates the power of modeling to improve our understanding of biological systems.

## Introduction

Insulin is the primary hormone in control of whole body energy metabolism in human beings. The hormone is secreted to the blood circulation by the *β*-cells, located in the islands of Langerhans in the pancreas. The adipose tissue and the adipocytes are important targets for insulin control of energy metabolism. Failure of the adipocyte and other target cells to properly respond to insulin, insulin resistance, is often associated with obesity and is a distinguishing feature of type 2 diabetes.

Insulin controls cellular metabolism by binding to the insulin receptor (IR) at the surface of the cell (reviewed in [1]). In response to insulin-binding the intracellular *β*-subunits of the transmembrane receptor, which carry protein kinase activity, autophosphorylate on specific tyrosine residues. Thus autophosphorylated, the IR is active against a set of intracellular signal mediator proteins, in particular the insulin receptor substrate-1 (IRS1), which becomes phosphorylated on tyrosine residues. Phosphotyrosines in IRS1 are recognized by proteins, containing a SH2-domain, which by binding to phospho-tyrosine become activated to transduce the insulin signal further downstream. The signaling eventually affects cellular metabolism, for example through an increase of glucose uptake or inhibition of lipolysis. Many of the downstream intermediary steps in the insulin signaling network of the target cells remain unidentified. However, also apparently well-characterized early aspects of insulin signal transduction remain incompletely understood and may thus also reveal novel features of importance for insulin action, both in normal and in disease states.

At the plasma membrane of adipocytes, the IR has been shown to be localized in plasma membrane microdomains, invaginations of the membrane, referred to as caveolae [2]. It is important that in human fat cells, but for instance not in rat adipocytes, the IRS1 is co-localized with the IR in caveolae [3]. In conjunction with insulin-binding the IR is internalized by endocytosis [4],[5], but the function of IR endocytosis has not been demonstrated. It may be to turn off signaling, e.g., by dephosphorylation of the receptor, by downregulating the number of IRs at the cell surface, or by clearing insulin from the circulation. Conversely, endocytosis may be a part of the signal transduction per se, e.g., by gaining access to downstream signaling intermediates or by providing for compartmentalization of the signaling. It has not yet been possible to determine experimentally which of these alternatives that are of highest importance, at the various time-scales involved in the signaling [6]–[15]. Conversely, the insulin-controlled internalization of IR has been shown to depend on IR autophosphorylation [13],[16],[17], but to be independent of downstream activation of IRS or phosphatidylinositol-3 kinase [17].

To gain further insight into which mechanisms that are most active during the early events of insulin signaling, we have measured the transient phosphorylation of IR and IRS1 during the first ten minutes after a step increase in extracellular insulin concentration. The mechanistic explanation to such transient data is typically not evident from a mere inspection of the time courses. Nevertheless, such data contains valuable information on the active mechanisms in a complex system, and measurements of rapid transient responses is one of the most widely used methods for characterization of technical systems [18]. In such studies, the information in the data is typically extracted from the data using a model based hypothesis testing approach. Such an approach is different from the kind of large-scale gray-box modeling approaches that typically are used in systems biology studies. Two such related models are [19],[20], and large-scale gray-box models are in general characterized by the fact that many more interactions are included than can be tested from the existing data. Conversely, in the hypothesis testing tradition followed here, we do not include all known mechanisms in the models. This typically corresponds to setting parameters to zero in a comprehensive model, and a key question is whether the included mechanisms are sufficient, necessary, or not sufficient, to explain the data. This gives information on which mechanisms that may, must, and cannot be significantly active during the specific time-scale. Apart from the overall methodology, the work also makes use of several non-trivial theoretical results and methods that can be re-used in other analyses of signaling systems.

## Results

### Experimental Time-courses for Insulin Signaling in Human Adipocytes

**Figure 1:**
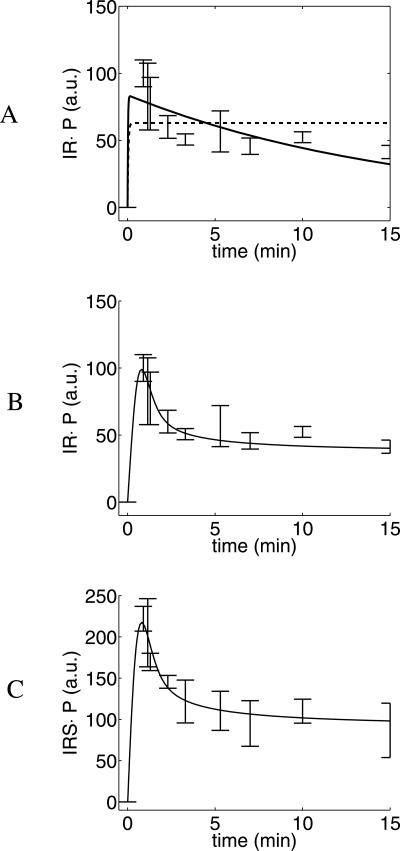
The experimental data and three representative model simulations. (A) shows experimental data for IR and their estimated standard deviations (vertical lines), and the agreement of a model without an overshoot (ℳ*_m,a_*, dashed line), and of a model with internalization and dephosphorylation but without recycling (ℳ*_i,c_*, solid line). (B) shows the same experimental data together with an acceptable model (ℳ*_i,a_*), and (C) shows the agreement between ℳ*_f_* and the IRS1 experimental data. Note that the experimental data has been normalised such that time-point zero has no standard deviation.

We examined the extent of phosphorylation of IR and IRS1 on tyrosine residues in human adipocytes. In three separate experiments, data were collected at 10 time points during 15 min, following a step increase from 0 to 0.1 *µM* in insulin concentration (Figure 1). The experimental set-up is limited to measurements of relative changes, i.e., all signals come with an unknown scaling factor. We measured phosphorylated and total IR and IRS1 by SDS-PAGE and immunoblotting. To achieve a robust measurement signal, the extent of phosphorylation of both IR and IRS1 were divided by total amount of IR and IRS1, respectively. The resulting signals are therefore proportional to the relative degree of phosphorylation of IR and IRS1. The rapid initial transient response was higher than the quasi-steady state level attained after about 5 min for both IR and IRS1 (Figure 1). This transient behavior is referred to as the overshoot in the data. The overshoot is clearly present both in each individual time course, and in their mean values. We now use a model based hypothesis testing approach, to translate these experimental observations to mechanistic insights.

### Mechanistic Hypothesis A: IR Dynamics Restricted to the Plasma Membrane

Three hypotheses are considered as possible mechanistic explanations to the observed overshoot. The first of these hypotheses, hypothesis A, assumes that the overshoot is generated by an interplay between the autophosphorylation and protein phosphatase activity at the plasma membrane only. It is interesting to consider the possibility whether such mechanisms might be the only ones significantly active in the IR signaling subsystem, since we are only considering the first few minutes of the response. The analysis shows that this possibility can be rejected based on the information in the collected data.

#### Models rejected by transfer function reformulations

**Figure 2:**
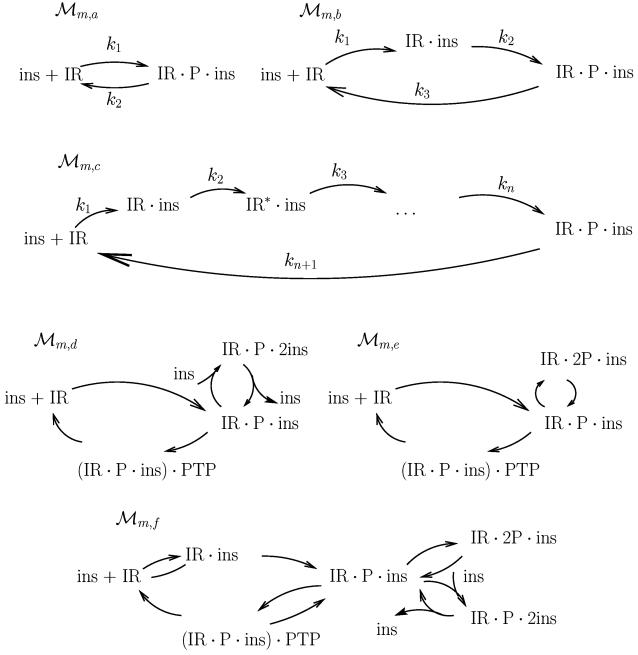
All models with only states at the membrane that can be rejected directly by lack of agreement, i.e., by a *χ* ^2^ test or by direct inspection of the properties of the transfer function. All these figures correspond to unique model structures, or sets of differential equations, as is explained in Text S1.

As explained above, a hypothesis like A does not correspond to a single model structure, but to a class of model structures, which we approximate through a large number of specific models belonging to the class. Almost all models do not have the ability to produce a sufficiently pronounced overshoot for any parameter value (i.e., even when disregarding realism). Examples of such model structures are depicted in Figure 2, and an explanation of how these figures may be translated into model structures is given in the Text S1.

The simplest of these model structures is ℳ*_m,a_*. The rejection may be concluded by reformulation into transfer function form and by application of Lemma 1 (derived in the Materials and Methods section). The same kind of rejection holds for ℳ*_m,b_*. The model structure ℳ*_m,c_* actually illustrates a class of model structures, even though it still is a sub-class of the model structures corresponding to hypothesis A. The common property in this sub-class is that the measured signal, the total concentration of phosphorylated insulin receptor, consists solely of the last state in a cyclic chain of intermediates before the return to the input state. All such model structures are unable to show an overshoot of the kind displayed prominently in the experimental data. Since the overshoot is clearly present in all three separate time-course experiments, we judge the inability of the models to display such an overshoot a sufficient reason for rejection.

The model structures ℳ*_m,d_*, ℳ*_m,e_* and ℳ*_m,f_* (Figure 2) may not be rejected by direct inspection of the transfer functions. These model structures include the possibility for multiple binding of insulin to the IR (ℳ*_m,d_*), the possibility for multiple tyrosine phosphorylations of IR (ℳ*_m,e_*), and the inclusion of complexes with the dephosphorylating phosphotyrosine protein phosphatase (ℳ*_m,f_*). These model structures are instead rejected by parameter optimization and a *χ* ^2^ test. The significance for rejection was chosen as 95%, and as can be seen in Table 1 (in Text S1) all models are clearly above the threshold. Many other models with only IR state variables at the membrane have been tested than those included in Figure 2. It should be noted that even though some of them can be argued to be biologically implausible, one should ideally test all model structures corresponding to hypothesis A before a conclusion can be made.

#### Models with the state (IR⋅ins)⋅PTP

There are some model structures corresponding to hypothesis A that may display a satisfactory agreement with the experimental data. We will now show that also these model structures may be rejected, through further model analysis and biochemical reasoning.

All discovered model structures corresponding to hypothesis A that give an acceptable agreement with the data include transient state variables such as (IR⋅ins)⋅PTP (insulin-bound IR in complex with the dephosphorylating phosphotyrosine protein phosphatase). The distinguishing feature of such state variables is that they describe a form of IR that has been dephosphorylated, but that is not yet a part of the original pool of IR.(IR⋅ins)⋅PTP describes the pool of IR that has been dephosphorylated, but that is still bound to the phosphatase PTP. Another such state could be IR⋅ins. Such an intermediate is, in principle, present in the cell and model structures that include this specific state are therefore not biochemically unrealistic per se.

However, (IR⋅ins)⋅PTP is believed to be transient, i.e., short-lived and therefore at any time only making up a small percentage of the total amount of IR (IR_tot_). This also holds for the intermediate state IR⋅ins, since IR is rapidly phosphorylated once bound to insulin. For a model of the IR subsystem to be realistic, it must therefore be able to describe the measured data and fulfill this additional criterion. Our analysis of the model, however, shows that it is impossible to fulfill this additional criterion while retaining a satisfactory agreement with the data.

The model analysis is based on the transfer function formulation of the model. Exact agreement with experimental data is not required, but only with the most prominent features of the data. The features of the experimental data that are chosen as initial requirements of the model are the ability to produce an overshoot and that the maximal value should be at least 50% higher than the steady state value. Since, in the collected experimental data, the maximal value is more than 100% higher than the steady state value and since the overshoot is clearly present in all three experiments and in the mean values, these requirements are believed to be firmly based on the experimental findings. A further requirement is that at least 35% of the original pool of receptors remain unphosphorylated. This is based on previous studies showing that more than 90% of the original pool of receptors remain unphosphorylated [14]. These requirements are included to narrow down the search in the parameter space and to provide sufficient requirements from the agreement with the data to be able to achieve the rejection. The chosen requirements are fast to check, e.g., compared to a global search based on an analysis of the cost function, and we search the parameter space using a grid. For all parameter that fulfill the two requirements, we pick the one that corresponds to the lowest steady state value of (IR⋅ins)⋅PTP, since we seek to know how small this variable can be, while still allowing for an acceptable agreement with the data.

**Figure 3:**
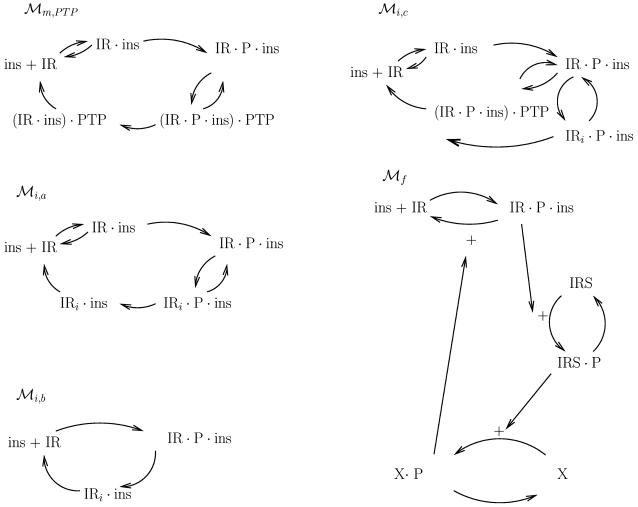
Models with a reasonable overshoot. The top left model structure only has states at the membrane, and that means that the last state before IR, (IR⋅ins)⋅PTP, will have a steady state value of at least 25% of the total receptor concentration. This is unrealistic and ℳ*_m,PTP_* is therefore rejected. ℳ*_i,a_*, ℳ*_i,b_*, and ℳ*_f_*, on the other hand, are accepted. All these figures correspond to unique model structures, or sets of differential equations, as is explained in Text S1.

We applied this approach to the model structure ℳ*_m,PTP_* (Figure 3), which contains reversible reactions in the initial binding to insulin (including the complex formation) and in the initial binding to PTP. The phosphorylation and dephosphorylation reactions, on the other hand, are believed to be essentially irreversible. Our experience from the addition of the multiple states of IR⋅P⋅ins, for instance those binding multiple insulin molecules, is that they do not drastically change the model predictions, but only distribute the IR⋅P⋅ins pool among more state variables. Therefore, ℳ*_m,PTP_* is judged to contain the most important degrees of freedom and to be representative for the assumption that only IR state variables at the membrane are important.

**Figure 4:**
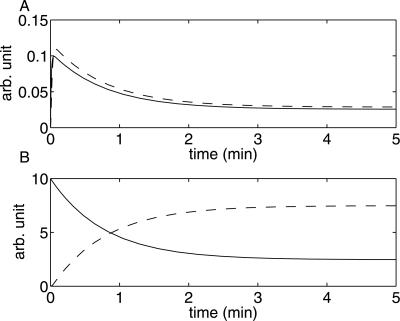
Some key simulations with the model structures ℳ*_i,a_* (or ℳ*_m,PTP_*). (A) Simulations of IR⋅P⋅ins (solid line) and IR*_i_*⋅P⋅ins (dashed). (B) IR (solid) and IR*_i_*⋅ins (dashed).

The outcome of the search for ℳ*_m,PTP_* was that the lowest possible steady state value of (IR⋅ins)⋅PTP, while still satisfying the above requirements, is more than 25% of the total amount of receptors, IR_tot_ (time-series for all state variables of ℳ*_m,PTP_* are included in Figure 4). This therefore violates the additional requirement on (IR⋅ins)⋅PTP, and thus rejects ℳ*_m,PTP_*. As this model structure describes a general model with (IR⋅ins)⋅PTP, and since many other model structures are special cases of this structure, this makes it plausible that all model structures with (IR⋅ins)⋅PTP should be rejected. Further, since all other model structures corresponding to hypothesis A are rejected due to lack of agreement with data, the entire class is thus rejected. Our conclusion is therefore that hypothesis A does not provide a satisfactory explanation for the experimental data set.

### Mechanistic Hypothesis B: Additional Inclusion of Endocytosis

#### An acceptable minimal model

The above analysis shows that some more mechanisms than IR dynamics at the membrane must be significantly active already during the first few minutes of the response. A potential such mechanism is internalization. Recall that the models with (IR⋅ins)⋅PTP are rejected exclusively due to the interpretation of the state variables, and that the model structure as such is capable of explaining the available data. For this reason, a model with the same graphical structure, but with a different interpretation of the state variables, might be acceptable. Such an acceptable interpretation is available when receptor internalization is included. This is exemplified by ℳ*_i,a_*. The two model structures ℳ*_m,PTP_* and ℳ*_i,a_* are identical from a graphical point of view, but with different interpretation of two of the nodes: the state (IR⋅P⋅ins)⋅PTP is replaced by an internalized form of the IR⋅P⋅ins, IR*_i_*⋅P⋅ins, and the state (IR⋅ins)⋅PTP is replaced by a dephosphorylated and internalized IR, IR*_i_*⋅ins. The analysis for ℳ*_m,PTP_* is of course equally applicable to ℳ*_i,a_*. This means that the state IR*_i_*⋅ins will have a steady state value of at least 25% of the total receptor concentration. However, while this is unrealistic for the short-lived state (IR⋅ins)⋅PTP, it is quite possible that the state IR*_i_*⋅ins has such a high steady state concentration at maximal insulin stimulation of the cells. Since this removes the only unrealistic feature of ℳ*_m,PTP_*, this means that ℳ*_i,a_* is our first non-rejected model structure.

The model structure ℳ*_i,a_* is acceptable (see Table 1 in Text S1), but it is not minimal. The two phosphorylated states are typically highly correlated and may thus be lumped into one state. Similarly, the formation of the intermediate state during the phosphorylation may be skipped (corresponding to a lumping of the reactions), as may the reversibility of the reactions. This gives the model structure ℳ*_i,b_*. Simulations with this model are in close agreement with the experimental data (Figure 1B). We have not been able to reduce this model further and it is thus our suggestion for a minimal acceptable model.

#### Rejection of models without recycling

The above results means that hypothesis B do provide for a satisfactory explanation of the experimental data set (note that it is sufficient with a single acceptable model structure to show that). However, our analysis indicates that hypothesis B, inclusion of endocytosis, is only an acceptable explanation if both internalization and recycling of IR to the plasma membrane are included. We show this too by a hypothesis testing approach.

A rather general model structure that includes internalization and dephosphorylation of IR at both the plasma membrane and in the cytosol is given by ℳ*_i,c_* (Figure 3). This model structure is similar to ℳ*_i,a_*, with the only important exception being that ℳ*_i,c_* does not have any recycling of the dephosphorylated receptor. ℳ*_i,c_* may display an overshoot, and the best possible agreement with the experimental data is shown in Figure 1A. The agreement is not as good as that for the models with recycling, but the question is if it is a statistically significant difference, given the uncertainty in the experimental data.

First we tested this using a *χ* ^2^ test. This shows that the lack of agreement in itself is sufficient for rejection with a significance of more than 95%. However, because the model does provide an overshoot, but of a different nature, it is interesting to do another statistical analysis as well. We therefore did a second test using the Akaike Information Criterion (AIC). The AIC value is 5.4 for ℳ*_i,c_*, and 3.5 after a recycling reaction has been added. This shows that the addition of a recycling reaction to ℳ*_i,c_* yields a better model, even when accounting for the general improvement of an additional parameter (Text S1). Yet another way to analyze this question would be to use likelihood ratio testing. However, since the problem does not fulfill the standard conditions, approximations (e.g. via bootstrapping) have to applied [21], and the approach is not pursued here. In any case, since ℳ*_i,c_* is a rather general model structure, we conclude that internalization and dephosphorylation alone do not provide a satisfactory explanation of the experimental data. This means that if no other mechanisms are significantly active (e.g., feedbacks from downstream signaling as described below), the observed overshoot and the following analysis has provided strong evidence that both internalization, intracellular dephosphorylation, and recycling of IR occur at significant levels already during the first few minutes of insulin stimulation.

### Mechanistic Hypothesis C: Alternative Addition of Feedback from Downstream Intermediates

While hypothesis B is like A with the additional possibility of endocytosis, hypothesis C is also like A but with the additional possibility of feedbacks from downstream signaling intermediates. There exist, indeed, in the literature suggestions of feedback from downstream signaling intermediates to, e.g., the phosphotyrosine protein phosphatase activity [22]–[25]. We here suggest an archetypical version of such a feedback, to show that hypothesis C also provides an acceptable explanation of the data. The suggested model structure (ℳ*_f_*; Figure 3) includes activation of IRS1 and its subsequent activation of X, which refers to some non-identified downstream signaling intermediate. The notation X is chosen in order to illustrate the fact that it is impossible to conclude without further experiments which specific feedback that is most likely to generate the observed behavior in the experimental data, and only that any feedback of the given character would be sufficient. The feedback to PTP illustrates the archetypical feedback, which also could be illustrated by a direct feedback to the IR, by for instance its serine phosphorylation [26]. The agreement between this model structure and the data is just as convincing as that for the minimal model ℳ*_i,b_*. Since it is sufficient that a single model structure from a given class produces a satisfactory explanation, in order for the whole class to be acceptable, we have now shown that hypothesis C is an alternative explanation to hypothesis B.

## Discussion

This paper has two parts. The first part reports a rapid overshoot in IR and IRS1 phosphorylation upon insulin stimulation of human fat cells. These observations, although interesting in themselves, do not provide any mechanistic insights by themselves, and mere inspection and reasoning around the data is not sufficient to evaluate which mechanisms that may and may not explain the given data in a satisfactory manner. The second part of the paper analyzes three biologically realistic and plausible mechanistic explanations: (A) direct phosphorylation and dephosphorylation of IR at the plasma membrane only; (B) the additional possibility of IR endocytosis; (C) the alternative additional possibility of feedback to IR from downstream intermediates. Our analysis has shown that A is not a satisfactory explanation, that B provides such an explanation if both internalization and subsequent recycling are included, and that hypothesis C provides such an explanation.

The mechanistic insights obtained here are the result of model based hypothesis testing and there are some important properties of such studies that should be pointed out. In a hypothesis testing framework, the most interesting result is when a model may be rejected. A rejection is also the kind of conclusion that is hardest to achieve. Ideally, all parameter values in all model structures belonging to the class of model structures corresponding to the tested explanation should be evaluated before a rejection has been shown. Conversely, evidence of the sufficiency of a mechanistic explanation is shown already by the existence of a single model structure at a single parameter point which gives a satisfactory agreement. Further, a model rejection is a strong statement since it will not be altered when new data are collected (unless, of course, the new data would point to errors in the previous data). The conclusions drawn here are thus not typical model predictions to be tested in validation experiments, but evaluations of possible mechanistic explanations for a given data set.

The significance of this modeling approach becomes evident when comparing with a previous modeling work by Sedaghat *et al.* [20]. That model structure is an example of a large-scale mechanistically detailed model for insulin signaling, and it includes both internalization of the insulin receptor and feedback effects from downstream metabolic intermediates to IRS1. Interestingly, the feedback signals do generate an overshoot in IRS1 phosphorylation. However, the Sedaghat model does not predict an overshoot in the IR phosphorylation, and must generally be revised to serve as a (single) explanation to our experimental data [27]. More importantly, however, the single model structure in [20] was evaluated at a single parameter point, and [20] is therefore a qualitatively different type of study than ours. A main drawback of such purely forward-simulation based studies is that most parameter values are unknown, especially *in vivo*. Analysis at a single parameter point is of course problematic if the chosen parameter values are unrealistic (which is the case for instance for the internalization constant in [20]). However, also if all parameter values are realistic, one does not know which model predictions and parameter values (i.e. active mechanisms) are necessary consequences of the given data and model structure, and which model predictions are merely outcomes of more or less arbitrarily chosen parameter values. An example of a stronger model prediction is for instance that for ℳ*_m,PTP_* herein, which says that all parameter values that give an acceptable agreement with our experimental data must also give a steady state concentration of (IR⋅ins)⋅PTP larger than 25%. Finally, it should be noted that not even an ordinary model rejection, reporting a lack of agreement with the data, may be done without global searches among all realistic parameter values.

There are also other related works. Interestingly, a transient overshoot in the phosphorylation of internalized IR has been reported [14]. However, that work did not provide any mechanistic explanations. A simulated model of signaling by the epidermal growth factor (EGF) receptor has been found to exhibit a transient phosphorylation overshoot when endocytosis of the receptor is included in the model [19]. However, the EGF receptor has a different mechanism of activation than IR, and there does not exist a thorough hypothesis testing approach that evaluates which mechanisms that may, and may not, produce such an overshoot. In a more recent time-course modeling of IR phosphorylation and endocytosis in Fao hepatoma cells [28], no transient phosphorylation overshoot was included, neither in the experimental data, nor in the model. Further, the authors used the Akaike Information Criterion (AIC) hypothesis testing approach to distinguish between all possible model structures. The AIC simply chooses a model as the best one, by weighting model agreement against number of parameters. This means that AIC does not provide any statistical measure on whether any of the evaluated models show an acceptable agreement with the data, or what the statistical significance of the conclusions are. That means that the AIC test alone would not have been sufficient to find the main conclusions and rejections provided in this article.

Our statistical testings are based on a number of assumptions. For instance, the noise in the system is approximated by white and Gaussian signals appearing exclusively in the measurements. This means that intrinsic system noise has been neglected, as have the indications that experimental noise from immunoblotting might be log-normal. To compensate for this limited complexity of the noise model, the variance of the noise has been exaggerated, and for many analyses only the most prominent features of the data (primarily the overshoot) have been used for the rejections.

Other limitations in our assumptions are due to our usage of ordinary differential equations (ODEs). This means that stochastic effects from individual particles, or individual cells, and subtle spatial phenomena (everything besides the internalisation itself) all are disregarded. These approximations have been judged acceptable since the available data do not allow for a more detailed inspection of the processes. It will be an important step forward in our understanding of these processes when we can measure data containing spatially resolved single cell data, and when we can more realistically describe processes in micro-environments such as caveolae, where IR and IRS1 are situated. So far, we can only speculate what the corresponding conclusions might be in such studies. For instance, the number of IR proteins per fat cell has been estimated to >2×10^5^ [29], and this should, according to generic studies such as [30], mean that molecular stochastic effects are insignificant, at least if the assumption of fast diffusion within the cell is valid. However, when it comes to incorporating the caveolae micro-environment properties, the fundamental kinetics will probably change (see e.g. [31]), and we have to-date no good guidelines for how such generalisations change the properties of a system.

In any case, despite these limitations, statistical assessments of the degree of uncertainty underlying model rejections do provide more detailed and objective statements than those based on simulations and/or subjective judgments alone. Most importantly, we have been able to draw mechanistic insights from a given set of time-series data; these mechanistic insights could not have been drawn using only classical biochemical reasoning.

## Materials and Methods

### Subjects

Samples of subcutaneous abdominal fat were obtained from female patients at the University Hospital of Linköping. Patients with diabetes were excluded. Pieces of adipose tissue were excised, during elective abdominal surgery and general anesthesia, at the beginning of the operation. The study was approved by the Local Ethics Committee and participants gave their informed approval.

### Materials

Rabbit anti-insulin receptor *β*-chain polyclonal and mouse anti-phosphotyrosine (PY20) monoclonal antibodies were from Transduction Laboratories (Lexington, KY, USA). Rabbit anti-IRS1 polyclonal antibodies were from Santa Cruz Biotech. (Santa Cruz, CA, USA). Insulin and other chemicals were from Sigma-Aldrich (St. Louis, MO, USA) or as indicated in the text.

### Isolation and Incubation of Adipocytes

Adipocytes were isolated by collagenase (type 1, Worthington, NJ, USA) digestion as described [32]. At a final concentration of 100 *µ*l packed cell volume per ml, cells were incubated in Krebs-Ringer solution (0.12 M NaCl, 4.7 mM KCl, 2.5 mM CaCl_2_, 1.2 mM MgSO_4_, 1.2 mM KH_2_2PO_4_) containing 20 mM Hepes, pH 7.40, 1% (w/v) fatty acid-free bovine serum albumin, 100 nM phenylisopropyladenosine, 0.5 U/ml adenosine deaminase with 2 mM glucose, at 37C on a shaking water bath. For analysis after 20–24 h incubation, cells were incubated at 37C, 10% CO_2_ in the same solution mixed with an equal volume of DMEM containing 7% (w/v) albumin, 200 nM phenylisopropyl adenosine, 20 mM Hepes, 50 UI/ml penicillin, 50 *µ*g/ml streptomycin, pH 7.40. Before analysis cells were washed and transferred to the Krebs-Ringer solution. Cells were then incubated at 37C with 100 nM insulin for the indicated time period.

### SDS-PAGE and Immunoblotting

Cell incubations were terminated by separating cells from medium by centrifugation through dinonylphtalate. The cells were immediately dissolved in SDS and *β*-mercaptoethanol with protease and protein phosphatase inhibitors, frozen within 10 sec, and thawed in boiling water to minimize postincubation signaling modifications in the cells and protein modifications during immunoprecipitation [32]. Equal amounts of cells as determined by lipocrit, that is total cell volume, were subjected to SDS-PAGE and immunoblotting. After SDS-PAGE and electrotransfer membranes were incubated with indicated antibodies that were detected using ECL+ (Amersham Biosciences) with horseradish peroxidase-conjugated anti-IgG as secondary antibody, and evaluated by chemiluminescence imaging (Las 1000, Image-Gauge, Fuji, Tokyo, Japan).

By two-dimensional electrofocusing (pH 3–10) - SDS-PAGE analysis and immunoblotting against phosphotyrosine and IRS1, >95% of the tyrosine phosphorylated 180-kD band was determined to represent IRS1 [33].

### Mathematical Modeling Using ODEs

In this paper we evaluate three mechanistic hypotheses for the explanation of experimentally observed phosphorylation dynamics. Each of these three hypotheses are too general to correspond to a single mathematical model that can make specific predictions, which can be compared with data. For this reason we consider classes of model structures in our analysis. In practise, these classes are approximated by a large number of specific models. In this paper we restrict ourselves to the consideration of models described by ODEs. The general form of an ODE is given by
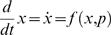
(1A)

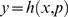
(1B)where *x*∈ℝ*^n^* is the n-dimensional column vector containing the state variables (concentrations denoted by a square bracket), *f* is a well-behaved (e.g., continuous and differentiable) function, *p*∈ℝ*^r^* contains the parameters, and *y* contains the measurement signals whose relation to the state variables and the parameters is given by the function *h*. Spatial transport in the form of endocytosis and recycling is described by the introduction of compartment specific state variables, where the subscript *i* denotes state variables that have been internalized. All the models are uniquely given by the figures according to standard interpretation of such figures; examples and more details are included in the Text S1.

### Reformulation into Transfer Function Form

Models are sought to be rejected in several different ways. The first way is rejection through analysis of a corresponding transfer function form. Transfer functions are commonly used for linear models [34], while the models considered here are nonlinear. Nevertheless, for the specific input studied (a step function), we can find equivalent linear models giving exactly the same responses, i.e., without approximations. This holds for all models accept ℳ*_f_*, and to see it on a more general level, consider the system
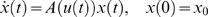
where *A*(⋅) is an ℝ*^n^* ^×*n*^-valued function, *x*(0)∈ℝ*^n^* is the state vector, e.g., concentrations of relevant substances, and *u*(*t*) is the input to the system. If *u*(*t*) changes from 0 to *u*_0_ at *t* = 0, the state vector *x*(*t*) will follow the same trajectory as for the system
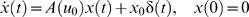
(2)where *δ*(*t*) is the Dirac function. In other words, we can study the impulse response of a linear system instead. Taking the Laplace transform of Equation 2 yields
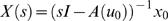
(3)


For instance, for ℳ*_m,a_* we have the following state-space description (derived in the Text S1)
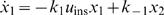
(4A)

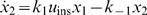
(4B)and the following transfer function description
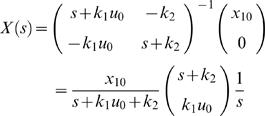
(5)with *X*(*s*) being the Laplace transform of 
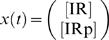
. Note the pole in *s* = 0, which means marginal stability. Biologically, this is due to the mass conservation, saying that [IR]+[IRp] is constant. For the same reason, all our considered model structures will contain a pole in *s* = 0.

Now, *X*(*s*) in Equation 5 can mathematically be interpreted as the step response of the transfer function
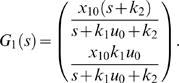
(6)


This allows us to transfer standard results from linear systems theory to our specific application.

We have derived two general results that allow for rejection by direct inspection of the transfer functions, i.e., without considering specific parameter values; these are presented in the following two subsections.

### An Overshoot Requires Zeros or Complex Poles

**Lemma 1** *Consider a stable, linear time-invariant system with transfer function G(s) having real poles and no zeros. Then, the impulse response of G(s) is positive for all* *t>0, i.e., the system cannot display an overshoot*.

*Proof.* Since *G*(*s*) has real poles, we can write *G*(*s*) as a cascade of first-order transfer functions *G_i_*(*s*), i.e.,
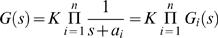
each with an impulse response
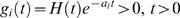
where *H*(*t*) is the Heaviside function, i.e., *H*(*t*) = 1 if *t*≥0, 0 otherwise. The lemma can now be proved by induction. Assume that 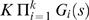 has a positive impulse response *y_k_*(*t*). Then the impulse response *y_k_*_+1_(*t*) for 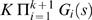 satisfies
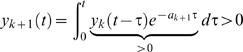
for all *t*>0.

Now, since the step response of *G*(*s*) can be obtained by integration of the impulse response, it follows that if *G*(*s*) has only real poles and no zeros, its step response is monotonously increasing, which means that no overshoot may occur.

For more details on conditions for positive impulse responses, see [35].

### The Last State Before an Input to a Cyclic Model Cannot Display Overshoots

Consider the system
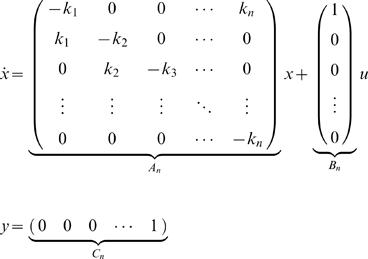



Assume that it is stable and has real poles. As described above the impulse response of this system is equivalent to the step response of the model ℳ*_m,c_*. The transfer function of the above system can be computed as
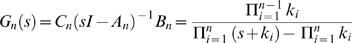



Note that the system has a pole at *s* = 0. Its impulse response equals the step response of
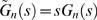



Since this transfer function has no zeros and real poles, its step response does not display any overshoot, according to Lemma 1. Therefore the final possibility would be that the overshoot in the models like ℳ*_m,c_* would be generated by non-real poles. However, this generates damped oscillations, and this is not seen in the data. Nevertheless, to be sure that no erroneous conclusions are drawn because of this interpretation of the data, also the first models in Figure 2 have been rejected by a *χ* ^2^ test.

### Evaluation of Models Through Optimization and Statistical Testing

For those models where a transfer function analysis is not sufficient for rejection, specific parameter values are needed: these are determined by parameter optimization. The resulting model is thereafter subjected to statistical tests, primarily *χ* ^2^ tests. Models can also be rejected if they are biochemically unrealistic in some other way, even though they show an acceptable agreement with the data. All models that can not be rejected in any of these ways are considered as acceptable explanations of the given data set. Further details on the parameter optimization and on the statistical testing are available in the Text S1. Finally, note that even though all models except for ℳ*_f_* may be analyzed using a transfer function study, this analysis gives a non-conclusive result for many more models than that, e.g., because the models may produce an overshoot, but it is unclear what its shape may be; for all those models we applied the more general optimization and statistical testing approach.

## Supporting Information

Text S1 Supplementary material and information about the methodology.
(0.06 MB PDF)
