# Supplementary material for: Model-Based Hypothesis Testing of Key Mechanisms in Initial Phase of Insulin Signaling
Source: PLoS Comput Biol. 2008 Jun 20;4(6):e1000096. doi: 10.1371/journal.pcbi.1000096 (PMC2424138; doi:10.1371/journal.pcbi.1000096)
Supplement: Text S1 — Supplementary material and information about the methodology. (0.06 MB PDF) [file pcbi.1000096.s001.pdf]

SUPPLEMENTARY MATERIAL

A Formulation in state-space form

We now exemplify how the model structures in this article may be constructed from the information contained in Figures 2 and 3. Consider the upper left model in Figure 2, $\mathcal{M}_{m,a}$. It consists of two state variables, describing the concentration of IR and IR $\cdot$ P $\cdot$ ins, respectively. Let these state variables be denoted x_1 and x_2. For all models appearing in the figures, except for $\mathcal{M}_f$, it is assumed that the reaction rates are given by mass-action kinetics. In mass action kinetics the reaction rate is given by a rate constant times the product of the concentrations of the substrates for the reaction. Let the rate constant for the upper and lower reaction be denoted k_1 and k_{-1}, respectively. Let the corresponding reaction rates be denoted v_1 and v_{-1}, respectively. Let the concentration of insulin be denoted u_{ins}, where the symbol u denotes the fact that insulin is an input to the model, and therefore not itself affected by the dynamics of the model. Insulin is assumed to be unaffected by the dynamics in the model, an assumption which is supported by the fact that insulin is situated in a much larger extracellular buffer. With these notations the reactions in $\mathcal{M}_{m,a}$ are given by

$$v_1 = k_1 u_{\text{ins}} x_1 \tag{7}$$

$$v_{-1} = k_{-1} x_2 \tag{8}$$

The reaction rates are used to form the differential equations. For a state variable corresponding to a concentration of a substrate, the reaction rate is subtracted from the right hand side of the differential equation, and if the state variable is a product it is added. This means that the differential equations (1a) for $\mathcal{M}_{m,a}$ are given by

$$\dot{x}_1 = -v_1 + v_{-1} \tag{9a}$$

$$\dot{x}_2 = v_1 - v_{-1} \tag{9b}$$

The measurements are always given by the sum of the state variables corresponding to a form of IR with a phosphate group, times an unknown scaling parameter denoted k_y. For $\mathcal{M}_{m,a}$ there is only one such state, and the measurement equation (1b) is therefore given by

$$y = k_y x_2 \tag{10}$$

Finally, at time zero it is assumed that all state variables are zero except for [IR], but the absolute value of [IR] at time zero is irrelevant for the analysis herein.

The above procedure can be generalized to all models structures in Figures 2 and 3 except for $\mathcal{M}_f$. This final model structure contains downstream signaling steps and regulated reactions, and both these features are lacking from the other model structures. The downstream signaling steps are treated in the same way as the IR step, where u_{ins} is replaced by the concentration of the relevant kinase. However, for the activation of the IR dephosphorylation reaction, there are some differences since X $\cdot$ P is not acting as a substrate to the reaction, but instead affecting the activity of the normal dephosphorylation. Here, this modification is formulated as a multiplicative term that is one in the case of no X $\cdot$ P, and increasing to a maximal activation as X $\cdot$ P goes to infinity. Let the

two parameters describing the maximal activation and the $X \cdot P$ concentration corresponding to half activation be denoted $k_{f,\max}$ and $k_{f,M}$, respectively. Then the dephosphorylation reaction, denoted by v_{-1}, is given by

$$v_{-1} = k_{-1}[\mathrm{IR} \cdot \mathrm{P} \cdot \mathrm{ins}](1 + \frac{k_{f,\max}[\mathrm{X} \cdot \mathrm{P}]}{k_{f,\mathrm{M}} + [\mathrm{X} \cdot \mathrm{P}]}) \tag{11}$$

where k_{-1} again denotes the normal reaction rate constant for the reaction. Note that there are many other more mechanistically based ways to form such a regulation. However, since the only purpose of this model structure is to show that such a regulation may give rise to the observed behavior, this simple phenomenological alternative is sufficient. For the same reason we neglect the possibility of effects from different volumes for IR/IRS1 and X [2]. The only model structure where such volume dependencies was important to check was $\mathcal{M}_{i,c}$, since this was the only model structure with internalization that was rejected. Our analysis, however, showed that the extra volume dependent parameter did not give an altered best agreement with the data.

Let the elements of the six-dimensional state vector x describe the concentrations of IR, $\mathrm{IR} \cdot \mathrm{P} \cdot \mathrm{ins}$, IRS, $\mathrm{IRS} \cdot \mathrm{P}$, X, and $\mathrm{X} \cdot \mathrm{P}$, respectively. Let the rate constants for the phosphorylation and dephosphorylation reactions for IR, IRS, and X, be denoted k_1, k_{-1}, k_2, k_{-2}, k_3, and k_{-3}, respectively. Then the differential equations (1a) for $\mathcal{M}_f$ are given by

$$\dot{x}_1 = -k_1 u_{\mathrm{ins}} x_1 + k_{-1} x_2 (1 + \frac{k_{f,\max} x_6}{k_{f,\mathrm{M}} + x_6}) \tag{12a}$$

$$\dot{x}_2 = k_1 u_{\mathrm{ins}} x_1 - k_{-1} x_2 (1 + \frac{k_{f,\max} x_6}{k_{f,\mathrm{M}} + x_6}) \tag{12b}$$

$$\dot{x}_3 = -k_2 x_2 x_3 + k_{-2} x_4 \tag{12c}$$

$$\dot{x}_4 = +k_2 x_2 x_3 - k_{-2} x_4 \tag{12d}$$

$$\dot{x}_5 = -k_3 x_4 x_5 + k_{-3} x_6 \tag{12e}$$

$$\dot{x}_6 = +k_3 x_4 x_5 - k_{-3} x_6 \tag{12f}$$

and the two measurements signals, y_1 and y_2, measuring phosphorylation of IR and IRS, respectively, are given by

$$y_1 = k_{y,1} x_2 \tag{13}$$

$$y_2 = k_{y,2} x_4 \tag{14}$$

where $k_{y,1}$ and $k_{y,2}$ are two unknown scaling parameters in the model. Again, the concentrations of the unphosphorylated state variables are assumed to contain all the concentration of the intermediate (IR, IRS or X) at time zero. Finally, all the model structures, and the files used to analyze them, are available from the authors on demand.

B Parameter estimation

For those models where a transfer function is not sufficient to draw conclusions about whether the model could be rejected, a combination of parameter estimation and statistical testing was used instead.

The parameter estimation approach is based on the classical least squares criterion. Consider again a general model structure of the form (1). Let the vector of measured signals at time t be denoted $y(t)$, and let the vector of simulated outputs corresponding to the parameters k, the unknown scaling parameters p_y, and the initial values x_0 be denoted $\widehat{y}(t|p)$, where p contains all the unknown parameters

$$p = (k, x_0, p_y) \tag{15}$$

Let the function describing the relation between the state variables x and the simulated output $\widehat{y}$ be denoted h

$$\widehat{y} = h(x, p) \tag{16}$$

This equation for $\mathcal{M}_{m,a}$ reads

$$y = p_y[\mathrm{IR} \cdot \mathrm{P} \cdot \mathrm{ins}] \tag{17}$$

where p_y is an unknown scaling parameter. Finally, let the weights quantifying the importance of each time point t_i and measurement signal y_j be denoted w_{ij}. With these notations the cost function to minimize is given by $V_N(p)$ as follows

$$V_N(p) = \sum_{i=1}^{N} \sum_{j=1}^{n_y} \left(\frac{y_j(t_i) - \widehat{y}_j(t_i|p)}{w_{ij}} \right)^2 \tag{18}$$

where N is the number of samples, n_y is the number of signals measured at each time-point. The estimated parameters, denoted $\widehat{p}$, are thus formally defined by

$$\widehat{p} := \arg\min_{p} V_N(p) \tag{19}$$

In practice, the estimation process is done via the optimization routines in the Systems Biology Toolbox for `MATLAB` [1]. The main algorithm used is a combination of Simulated Annealing, which is a global optimization method, and the local, but not gradient based, nonlinear simplex method. The weights are chosen as 1 or as the estimated variance at each time point. Note that this gives a higher weight to the first minutes in the time-series since more measurements were made in this time-period; this is good because most of the information in the data series is contained in this time-frame. Finally, for the models with a good, but not acceptable, agreement with the data, other weights were tried as well.

The number of iterations at each temperature were always at least 50*n_y, and the temperature always ranged from 1000 to 0 with changes by a factor of 10 at each change, and with a restarted local search at the last temperature. For the models with a fairly good, but not acceptable, agreement with the data more extensive searches were made. These more elaborate searches were made to minimize the risk that erroneous conclusions have been made because of an inadequate search in the parameter space. However, no such more elaborate search changed the final agreement with more than a single percent in terms of the cost function. More details regarding the parameter estimation, and examples of the scripts and limits used in the various searches are available from the authors on request.

C χ^2 tests

All statistical tests are based on a null hypothesis, H_0. The null hypothesis in our χ^2 tests is that the true data $y(t)$ have been generated by the given model output, $\widehat{y}(t|\widehat{p})$, for a specific realization of the measurement noise $v(t)$

$$H_0 : y(t) = \widehat{y}(t|\widehat{p}) + v(t) \quad \text{for all } t \tag{20}$$

If this was true, the residuals $\varepsilon(t)$, defined as the difference between the measured and simulated outputs, would have the same distribution as the measurement noise $v(t)$. In this work we assume that the measurement noise is generated by independent normally distributed processes with mean value zero, and with variances $\sigma_j^2(t)$ for the time point t and measurement signal j. The variances are estimated by the spread of the measurements from the individual experiments at the different time-points and measurement signals.

If the squared residuals are divided by the variance, the null hypothesis is thus that a squared normally distributed sequence is obtained. This means that the null hypothesis assumes that the following test quantity

$$\mathcal{T} = \sum_{i=1}^{N}\sum_{j=1}^{n_y}\left(\frac{\varepsilon_j(t_i|\widehat{p})}{\sigma_j(t_i)}\right)^2 = \sum_{i=1}^{N}\sum_{j=1}^{n_y}\left(\frac{\widehat{y}_j(t_i|\widehat{p}) - y(t_i)}{\sigma_j(t_i)}\right)^2 \tag{21}$$

should follow a χ^2 distribution. One may therefore evaluate (21) and compare it with a χ^2 distribution of appropriate order. In this way one may judge how unlikely it is that the null hypothesis is true. Here we choose a 95% limit for rejection. Finally, since we use the same data set both for the estimation and for the testing step, the degrees of freedom in the χ^2 distribution is $N \cdot n_y - r$, where r is the number of parameters varied in the estimation step (or the number of identifiable parameters if that is smaller). Here the number of identifiable parameters is 3 (estimated using an identifiability analysis in [2]), and we assume it to be the same for all model structures, since the number of identifiable parameters is primarily a property of the experimental data, and not of the model structure. This means that the degrees of freedom is 11-3 = 8 [2]. The resulting χ^2 test results for all the model structures in Figures 2 and 3 is given in Table 1.

D Akaike Information Criterion

The Akaike Information Criterion (AIC) is given by [3, 4]

$$\log((1 + 2\frac{r}{N}V_N)) \tag{22}$$

where r denotes the number of (identifiable) parameters, N the number of measurement points, and V_N the cost function. The cost function should be unweighted but scaled with the number of samples, i.e.,

$$V_N = \frac{1}{N}\sum_{k=1}^{N}(y(t_k) - \widehat{y}(t_k, \hat{p}))^2 \tag{23}$$

where y and $\widehat{y}$ denote the measured and simulated output, respectively. When models are compared the model with the lowest AIC value is chosen.

Model Structure	Test Value	Threshold (95%)	Acceptable agreement?
$\mathcal{M}_{m,a}$	58	15.5	No
$\mathcal{M}_{m,b}$	58	15.5	No
$\mathcal{M}_{m,c}$	58	15.5	No
$\mathcal{M}_{m,d}$	58	15.5	No
$\mathcal{M}_{m,e}$	58	15.5	No
$\mathcal{M}_{m,f}$	58	15.5	No
$\mathcal{M}_{i,a}$	3.8	15.5	Yes
$\mathcal{M}_{i,b}$	3.8	15.5	Yes
$\mathcal{M}_{i,c}$	32	15.5	No
$\mathcal{M}_{f}$	8.5	15.5	Yes

Table 1: The results of the χ^2-tests for the model structures in Figures 2 and 3.

REFERENCES

1. Schmidt, H., and Jirstrand, M. (2006) *Bioinformatics* **22,** 514-515

2. Cedersund, G. (2006) "Core-box modelling – theoretical contributions and applications to glucose homeostasis related systems,", Ph.D. thesis, Chalmers, Sweden

3. Akaike, H. (1974) *IEEE Trans. Autom. Control*, **AC-19,** 716-723

4. Akaike, H. (1981) In *Trends and Progress in System Identification*, Ed. P. Eykhoff, Pergamon Press, Elmsford, N.Y
